# On the Diagnosis of Anæmic Murmurs

**Published:** 1872-06

**Authors:** J. H. Hutchinson

**Affiliations:** Transactions of the College of Physicians of Philadelphia


*On the Diagnosis of Anæmic Murmurs.* By Dr. J. H. Hutchinson. (Transactions of the College of Physicians of Philadelphia.)

Although much has been written upon the subject of anæmia, and upon the means of distinguishing murmurs accompanying it from those produced by cardiac lesions, yet cases occasionally occur in the practice of every physician, in which it is almost if not quite impossible to determine from a single examination whether or not there exists organic disease of the heart. Such a difficulty constantly arises in cases of acute rheumatism, a disease in which there is a decided tendency, on the one hand, to the occurrence of inflammation of the membranes of the heart, and, on the other, especially if the case have been treated by antiphlogistic remedies, or by large doses of alkalies, to the production of anæmia, which may persist long after the patient is well enough to leave his bed, or even the house, and which may, in consequence of the existence of cardiac murmur, give rise to the impression that permanent injury has been inflicted upon the heart. It, therefore, frequently becomes a matter of some importance to be able to say whether the murmur indicates valvular trouble, or whether it is simply an attendant upon alterations in the composition of the blood. In spite, too, of all that has been written, the cause of these anæmic murmurs still remains undiscovered.

The definition of the anæmic murmur given by Walshe, the general accuracy of whose description will scarcely be impugned, is as follows:

"An intra-cardiac hæmic murmur of this variety is of moderate or very slight intensity, commonly of medium or low pitch, short or moderately prolonged, of whiffing quality, very easily rendered temporarily harsh by excitement of the heart, and *modified in intensity by certain changes in posture.** This murmur is, as far as I

* The italics are our own.

have observed, invariably basic in seat and systolic in time, produced at the orifices of the aorta and of the pulmonary artery—with a force at each, proportional to the power of its communicating ventricle; scarcely conducted along the aorta at all, frequently audible, on the contrary, at the second left or pulmonary cartilage; only in exceptional cases audible below the nipple; and never within my experience perceptible as far as the left apex."

He also attaches much importance to the fact that cardiac murmurs of this class are generally accompanied by the venous hum. In regard to the mechanism of these murmurs he says this is obscure enough, but that it seems impossible to ignore the influence of the composition of the blood "in highly marked spanæmia;" he says, "neither the flaccidity of the veins, pressure on their surface, nor even velocity of current, is required for the generation of murmur."

Other writers are disposed to attribute their production to augmented friction of the blood against the orifices of the aorta and pulmonary artery, and others to the diminished tension of the valves and vascular walls, which is caused by anæmia, and which permits vibrations in them to be more readily excited and maintained than in health. All, however, so far as I am aware, with a single exception, are agreed that the seat of their production is the aortic or pulmonary semilunar valve.

A paper will be found in the *Archives Generales* for 1866, in which the writer, M. Parrot, attempts to prove that they are really due to tricuspid regurgitation. This regurgitation is caused by an enlargement of the right auriculo-ventricular orifice, consequent upon dilatation of the cavity of the right ventricle. The walls of the right ventricle are thin in comparison with those of the left, and it is thought that when badly nourished, as they must be in anæmia, they oppose but a feeble resistance to the dilating influence of the blood. M. Parrot says that the so-called anæmic murmur is not heard immediately after a large hemorrhage, but sometime later, when the dilatation of the cavity has been produced. He says, moreover, that the seat of the greatest intensity of these murmurs is not, as has been generally supposed, at the pulmonary cartilage, but invariably over the body of the right ventricle, and generally at the level of the fourth rib, a little to the left of the sternum, and that they are constantly accompanied by pulsation of the jugular veins, which may be visible under ordinary circumstances, or may be only perceived when the patient is placed in bed with his head a little lower than his shoulders. It cannot be doubted that a murmur depending upon functional rather than organic disease is occasionally heard in the position above indicated, but there are few physicians accustomed to the careful examination of the heart, who will agree with M. Parrot that this is the most frequent seat of anæmic murmurs, or that these are constantly or even often attended by venous pulsation.

With the view of bringing to the notice of the profession a sign which I conceive to be of some importance in the diagnosis of anæmia, and which, if not wholly unknown, is at least practically ignored, I have determined to report the following cases, which are a few of those in which it has been observed.

CASE I. Annie C., æt. 18, born in Ireland, single, a domestic. Admitted into the Pennsylvania Hospital, November 17, 1870. Family history shows a tendency to phthisis. Has lost her mother and one sister, of consumption. Her health has generally been very good; when well, is stout and strong and has a good color. Came to America at the age of 15, and shortly afterwards began to "live out." Has lived in the same place during the past three years. Her work was hard, and she had sufficient though not very attractive food, and as a rule scarcely ever went out. She has never had malarial fever or rheumatism.

Began to menstruate when between 14 and 15 years of age, and the discharge continued at intervals of a month for a short time, but soon afterwards recurred regularly every fortnight, and has since continued to do so, when well. The discharge continued for from two to three days, and was usually abundant, necessitating frequent changes of napkins. Two years ago she became, without any apparent (additional) cause, very pale and weak, and her menses ceased entirely for two months. She lost flesh and strength, and was obliged to give up work. Took tincture of chloride of iron, and went into the country. This treatment soon restored her to health, and her menses reappeared. Last July, after unusually close confinement to the house and hard work, she lost appetite, became pale, felt weak, grew thin; had shortness of breath and palpitation on slight exertion, and her menses appeared only once a month, while their duration was a little extended. At this time she had frequent bleeding at the nose. Has never had cough, never hæmoptysis, and never any uncommon loss of blood save by the frequent menstruation. She took tincture of the chloride of iron without apparent benefit. She continued to lose color until admission.

Two weeks ago began to suffer from severe irregular pains, neuralgic in character, at the base of the chest on both sides anteriorly. Has never had leucorrhœa.

On admission she was markedly anæmic; the hands and face were white, the circulation of the surface sluggish, the lips and tongue pale, and the conjunctiva pearly, tongue clean, appetite poor, and her bowels constipated. She has no gastric or pulmonary symptoms. She sleeps well.

The urine is somewhat increased in quantity, but otherwise normal. Over the base of heart is heard a well-marked anæmic murmur. It is systolic in time, soft in quality, and is not heard at the apex. When the patient is placed in the recumbent posture, this murmur becomes much more intense, and this is evidently not due to increased action of the heart. The murmur becomes less intense when the patient again assumes the erect position. There is no diastolic murmur, and no reason to suspect any organic disease of the heart. At the sides of the neck, between the two insertions of the sterno-cleido mastoid muscle on the right side, is heard a continuous, musical, venous hum.

Dec. 23. Patient improved very much; her gums, which were so pale, have now a good color; the color has returned to her lips and cheeks; a soft murmur is still heard at the base of the heart.

Jan. 1, 1871. Condition still continues to improve; the soft murmur before remarked has never disappeared.

16th. Discharged, much relieved.

CASE 2. John P., æt. 16, born in England; occupation, sailor boy; admitted April 13, 1871. Family healthy; father died of a fever; previous health has always been good, and has been a sailor for about 17 months. Was taken sick on voyage from the West Indies; had fever, (?) general weakness, etc. Soon after his feet swelled, and he became unable to attend to his duties.

13th. Present condition anæmic and weak; feet and legs swollen, and pit slightly on pressure; appetite good; no symptoms of digestive trouble. A soft systolic murmur is heard at the pulmonary cartilage; by placing the stethoscope over either jugular, the "bruit de diable" can be detected. Urine was examined, but neither casts nor albumen were found. Passes urine in normal quantity; symptoms seem to be dependent upon the anæmia. He was ordered the infusion of scoparium and the citrate of iron and quinia in doses of five grains three times daily, and good diet.

19th. Œdema diminished considerably since admission. The infusion of scoparium was only given for a few days. The murmur before noted is heard most distinctly when the patient is in the recumbent posture.

29th. Has improved to a certain extent; has lately had an attack of bronchial catarrh which has thrown him back. There is some lingering œdema of the lower extremities, though not nearly so marked as upon admission. Treatment unchanged. Patient was subsequently discharged very much relieved, the murmur having entirely disappeared.

CASE 3. Annie G., æt. 19, American, domestic, admitted into Pennsylvania Hospital Oct. 12, 1871. Family of patient seems to be healthy. She began to live out four years ago. Her work has generally been light; her food has always been good; she has never had anxiety of any kind; she began to menstruate at 15, and was always perfectly regular until one year and a half ago; she at that time missed one or two periods, and has ever since menstruated very irregularly. Soon after this functional derangement she became, as she expresses it, nervous; had headaches and backaches, and worried about herself. She gradually lost color. The pallor of her skin progressed steadily, but her strength held out tolerably well until a few weeks before her admission to the hospital.

Condition on admission. Exceedingly anæmic; skin presents almost a waxy appearance; mucous membrane of lips and mouth white; appetite poor; bowels regular. Suffers from palpitation of the heart on exertion, a very faint hæmic murmur is heard at pulmonary cartilage; this is rendered considerably more distinct when patient is placed in the recumbent posture. Ordered the best diet that the hospital affords, and a teaspoonful of the following mixture:

Liq. Potass. Arsenit., - - - fl. dr.ij;
Tr. Nucis Vomicæ, - - - - fl. oz.ss;
Vini Ferri Amari, - - - fl. oz.viiss.
To be taken three times daily.

Nov. 6. Her face has gained some little color; is somewhat stronger, but improvement on the whole has been slow. A faint venous hum is heard over the jugular veins, but it is only heard when slight pressure is made with the stethoscope. The cardiac murmur is heard not merely at the pulmonary cartilage, but when the patient is lying down it is heard over the body of the heart and along the left edge of the sternum as far down as the ensiform cartilage.

In all these cases it will be noticed that the murmur became more intense upon the patient assuming a recumbent position; in one case, indeed, it was occasionally only to be heard in this position, and in this case the venous hum was to be heard only when pressure was made with stethoscope over the course of the jugular vein, and the head of the patient was turned strongly to one side, so as to put the muscles of the other side on the stretch. I am, in consequence, inclined to believe, that, in many cases in which the anæmia is not marked, and in which no murmur can be heard when the patient is standing, it may sometimes be perceived by causing the patient to change his position. It will be recollected that in the passage quoted from Walshe's work on *Diseases of the Heart*, he calls attention to the fact that the anæmic murmur is modified in intensity by certain changes of position, but the context justifies the inference that these changes are of a nature to cause some excitement of the heart's action; whereas, in the cases reported by me, the change from the erect to the recumbent posture did not cause increased frequency of the pulse, which was, of course, noted carefully before and after having recourse to auscultation. The patients were also re-examined when they had risen from the bed, and in all these cases the murmur was then found to be less intense than it had been the moment before.

In a search through all the works on physical diagnosis which are at present accessible to me, I find but a single reference to this important point in the diagnosis of anæmia. And this is by an author whose book is indeed a standard one, but is also not as much read as it deserves to be. I allude to Dr. Stokes, of Dublin. When speaking of the murmur occasionally heard during the convalescence from maculated typhus, he mentions, as one of its characteristics, its frequent disappearance or diminution in the erect position, and says: "This may help us in distinguishing it from an organic murmur, but I am unable to say whether the same is not observable in ordinary cases of anæmia."

I am at a loss to explain the cause of the increased intensity of the murmur in the recumbent position. It is possible that the relation of the heart to the large arteries at its base is of such a nature that increased friction of the blood against the sides of the arterial orifices takes place in this position; or it may be, that, the respiration being less free and more embarrassed in this than in the erect position, the circulation through the pulmonary vessels is interfered with, and, as a consequence, that a murmur may be produced at the pulmonary cartilage; or, admitting M. Parrot's theory to be correct, that this interference with the pulmonary circulation favors the occurrence of tricuspid regurgitation.

